# The effect of the antisickling compound GBT1118 on the permeability of red blood cells from patients with sickle cell anemia

**DOI:** 10.14814/phy2.14027

**Published:** 2019-03-27

**Authors:** Halima Al Balushi, Kobina Dufu, David C. Rees, John N. Brewin, Anke Hannemann, Donna Oksenberg, David C.‐Y. Lu, John S. Gibson

**Affiliations:** ^1^ Department of Veterinary Medicine University of Cambridge Cambridge United Kingdom; ^2^ Global Blood Therapeutics South San Francisco California; ^3^ Department of Paediatric Haematology King's College London School of Medicine King's College Hospital NHS Foundation Trust London United Kingdom

**Keywords:** Sickle cell anemia, GBT1118, O_2_ affinity, sickling, potassium permeability, cell volume, hemolysis

## Abstract

Sickle cell anemia (SCA) is one of the commonest severe inherited disorders. Nevertheless, effective treatments remain inadequate and novel ones are avidly sought. A promising advance has been the design of novel compounds which react with hemoglobin S (HbS) to increase oxygen (O_2_) affinity and reduce sickling. One of these, voxelotor (GBT440), is currently in advanced clinical trials. A structural analogue, GBT1118, was investigated in the current work. As RBC dehydration is important in pathogenesis of SCA, the effect of GBT1118 on RBC cation permeability was also studied. Activities of P_sickle_, the Gardos channel and the KCl cotransporter (KCC) were all reduced. Gardos channel and KCC activities were also inhibited in RBCs treated with Ca^2+^ ionophore or the thiol reagent *N*‐ethylmaleimide, indicative of direct effects on these two transport systems. Consistent with its action on RBC membrane transporters, GBT1118 significantly increased RBC hydration. RBC hemolysis was reduced in a nonelectrolyte lysis assay. Further to its direct effects on O_2_ affinity, GBT1118 was therefore found to reduce RBC shrinkage and fragility. Findings reveal important effects of GBT1118 on protecting sickle cells and suggest that this is approach may represent a useful therapy for amelioration of the clinical complications of SCA.

## Introduction

Sickle cell anemia (SCA) represents one of the commonest severe inherited disorders affecting millions of people worldwide (Piel et al. 2013). The etiology is well known – in most cases a single amino acid substitution at the sixth position of the *β* chain of Hb with valine replacing glutamic acid produces HbS rather than the normal adult HbA (Rees et al. 2010). About two‐thirds of cases are homozygous for HbS (HbSS genotype) while about one‐third are heterozygous for HbS and HbC, in which the *β*6 amino acid substitution is lysine instead of valine. There are, in addition, a number of other rarer genotypes (Rees et al. 2010).

The pathogenesis of SCA is complex but the precipitating event is the ability of deoxygenated HbS molecules to adhere to each other (Steinberg 1999). The resultant polymers are highly organized with 14‐stranded helices of insoluble HbS, sufficiently rigid to alter RBC shape – the sickling shape change – distorting the normal biconcave morphology into sickles and other bizarre shapes. The consequence is sticky, fragile RBCs with greatly reduced life‐span and poor rheological features. Chronic anemia ensues. In addition, sickle cells also occlude small blood vessels with symptoms of ischemia and organ damage, including pain, strokes, nephropathy, osteonecrosis, dactylitis, leg ulcers, and priapism. Notwithstanding, the clinical picture is markedly variable between patients with some mildly or even subclinically affected, while others present with much more severe complications requiring frequent hospitalization and support for multiple organ damage (Steinberg 1999; Rees et al. 2010). In most cases, it is not known why the clinical severity has a variable course.

Despite its high incidence worldwide, treatment remains largely supportive (Rees et al. 2010). Blood transfusion, neonatal vaccination, and provision of antibiotics have all been useful in reducing mortality but specific therapies remain inadequate. Hydroxyurea has shown some success (Platt et al. 1984; Charache et al. 1987). This reagent appears to work by increasing the expression of fetal Hb (HbF), which dilutes RBC HbS and inhibits polymer formation. Hydroxyurea is not without its problems, however, being potentially teratogenic and showing a variable response between patients (Rees 2011). There has therefore been considerable effort at discovering new therapies (Gibson et al. 2015).

A particularly fruitful approach has been the design of adducts which interact directly with HbS and reduce its tendency to polymerize. These increase the oxygen affinity of HbS and “left shift” the Hb oxygen equilibrium curves (OECs) (Safo and Kato 2014). Since their inception in the 1970s, a large number of compounds have been tested including BW12C (Fitzharris et al. 1985) and tucaresol (Rolan et al. 1995). Until recently, the most promising was 5‐hydroxymethyl‐2‐furfural (5HMF or Aes‐103; Abdulmalik et al. 2005) but none progressed to phase 3 clinical trials until the design of voxelotor (also known as GBT440) by Global Blood Therapeutics (Lehrer‐Graiwer et al. 2015; Dufu et al. 2016; Oksenberg et al. 2016; Metcalf et al. 2017; Howard et al. [Link]). Voxelotor increased the HbS O_2_ affinity, has excellent pharmacokinetic properties, reduces in vivo sickling, is well tolerated, and is currently in phase 3 clinical trials. GBT1118 is a structural analogue of voxelotor with similar pharmacological properties (Dufu et al. 2017).

An important feature of RBCs from SCA patients is their increased cation permeability (Lew and Bookchin 2005). As a consequence, cells lose KCl and osmotically obliged water with subsequent RBC shrinkage and elevation of the concentration of intracellular HbS ([HbS]). Clinically, this is particularly significant as the lag time to polymerization is inversely proportional to [HbS]^15‐30^ (Eaton and Hofrichter 1987). A small shrinkage means that cells are markedly encouraged to undergo sickling as they traverse hypoxic regions of the microvasculature. Three transport systems are predominantly involved (Lew and Bookchin 2005): a deoxygenation‐induced cation conductance sometimes called P_sickle_, a Ca^2+^‐activated K^+^ conductance or Gardos channel, and the KCl cotransporter (KCC). P_sickle_ is activated by HbS polymerization and RBC shape change, the Gardos channel by a rise in intracellular [Ca^2+^], while KCC is modulated by pH, volume, urea, and oxygen via cascades of conjugate protein kinases and phosphatases (Gibson and Ellory 2003; Lew and Bookchin 2005). The Gardos channel is encoded by *KCNN4* (Hoffman et al. 2003), while there are three isoforms of the KCl cotransporter found in RBCs, KCC1, 3, and 4, encoded by the genes *SLC12A4, 6 & 7* (Pan et al. 2011; Arroyo et al. 2013). The molecular identity of P_sickle_, however, remains elusive although a possible involvement of PIEZO1 has been proposed recently (Zarychanski et al. 2012; Gallagher 2017).

Understanding the mechanisms involved in KCl loss and uncovering potential inhibitors have represented an additional therapeutic approach. Over the years, potential inhibitors to all three transport systems have been investigated (see table 1, Gibson et al. 2015). A particularly promising compound was the clotrimazole analogue ICA17043 known as Senicapoc (Ataga et al. 2008; Ataga and Stockler 2009). Senicapoc inhibits activity of the Gardos channel in vitro and thereby reduces solute loss and shrinkage. In SCA patients in vivo, Senicapoc was also effective in increasing RBC hydration. Episodes of pain, however, were not reduced and this agent was discontinued (Ataga et al. 2008), perhaps prematurely. The search for more effective compounds continues.

Here, we describe the in vitro effects GBT1118 on RBCs from SCA patients. We confirmed a marked increase in O_2_ affinity and reduction in RBC sickling. In addition, the reagent also inhibited all three dehydrating pathways in sickle RBCs, with increase in RBC hydration, and a reduction in RBC fragility. These findings are particularly exciting as they suggest that GBT1118 may have multiple actions, reducing sickling by both increasing oxygen affinity and also by maintaining a lower intracellular concentration of HbS, and stabilizing the RBC membrane.

## Materials and Methods

### Chemicals

GBT1118 was synthesized and provided by Global Blood Therapeutics (South San Francisco, CA). Clotrimazole and A23187 were purchased from Calbiochem (Nottingham, UK). ^86^Rb^+^ was supplied by Perkin Elmer (Beaconsfield, UK) and nitrogen by BOC (Guildford, UK). Other chemicals were purchased from Sigma Chemical Co. (Poole, Dorset, UK).

### Sample collection and handling

Blood samples for routine tests according to clinical indications were taken into the anticoagulant EDTA from patients homozygous (HbSS) for SCA, attending the sickle cell clinic at King's College Hospital. Once routine testing had been completed, discarded, anonymized blood was analyzed. The use of discarded blood was approved by the local ethics committee following guidelines set out in the Declaration of Helsinki. Samples were kept at 4 °C until use within 48 h.

**Figure 1:**
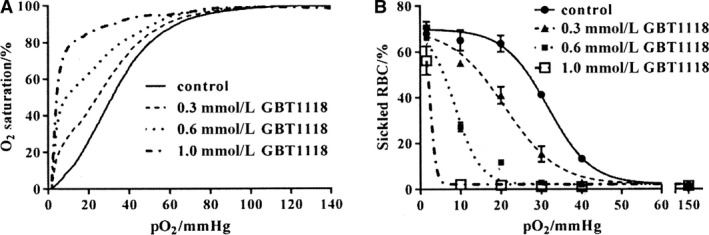
Effect of GBT1118 on O_2_ saturation and sickling of red blood cells (RBCs) from patients with sickle cell anemia (SCA). RBCs were preincubated without or with different concentrations of GBT1118 (0, 0.3, 0.6, and 1 mmol·L^−1^) for 60 min at 37°C at 20% hematocrit (Hct) after which O_2_ saturation and sickling were measured at O_2_ tensions of 150, 40, 30, 20, 10, and 1.6 mmHg. GBT1118 remained present throughout the assay. O_2_ saturation was measured spectrophotometrically using a Hemox analyzer. RBC aliquots were then removed from the sample chamber were fixed with 2% glutaraldehyde for morphometric analysis by light microscopy. Full details are given in the Methods. Data are from a single representative experiment.

Blood from a homozygous (HbSS) SCA patient obtained from the University of North Carolina [UNC, Chapel Hill, NC (IRB # 88‐034)] was used for RBC sickling and O_2_ affinity measurements (Fig. 1).

### Solutions and tonometry

The standard saline (Cl‐MBS) comprised (in mM): 145 NaCl, 1.1 CaCl_2_, 5 glucose, and 10 MOPS, pH 7.4 at 37°C, 290 ± 5 mosmol.kg^−1^ H_2_O. For experiments in which Cl^−^ dependence of K^+^ influx was examined, NO_3_^−^‐containing salts replaced those containing Cl^−^ (N‐MBS). To prevent the rapid RBC shrinkage which would otherwise occur following maximal stimulation of the Gardos channel in experiments in which intracellular Ca^2+^ was directly raised by incubation with the Ca^2+^ ionophore A23187, a high‐K^+^‐ and low‐Ca^2+^‐containing saline was used with Ca^2+^ buffered with EGTA, comprising (in mM): 80 KCl, 70 NaCl, 2 CaCl_2_, 0.15 MgCl_2_, 2 EGTA, 5 glucose, and 10 MOPS (HK‐MBS) with a free [Ca^2+^]_o_ of 10 *μ*mol·L^−1^. The high K^+^ saline prevents rapid shrinkage of RBCs which would otherwise occur following Gardos channel activation. The wash solution to remove unincorporated ^86^Rb^+^ comprised isotonic MgCl_2_ (107 mmol·L^−1^), buffered with MOPS (10 mmol·L^−1^), pH 7.4 at 4°C (Mg‐MBS). Stock solutions of bumetanide (10 mmol·L^−1^) were prepared in 100 mmol·L^−1^ Tris base and used at a final concentration of 10 *μ*mol·L^−1^. Stock solutions of ouabain (10 mmol·L^−1^) were prepared in distilled water and used at a final concentration of 100 *μ*mol·L^−1^. Stocks of clotrimazole (CLT; 5 mmol·L^−1^) were prepared in DMSO and used at a final concentration of 5 *μ*mol·L^−1^. Whole blood was washed five times in N‐MBS to remove Cl^−^, plasma, and buffy coat. RBC suspensions were then preincubated at 20% hematocrit (Hct) in eppendorf tubes with or without GBT1118 (up to 3 mmol·L^−1^ GBT1118, dissolved in DMSO – untreated RBCs were exposed to the same final concentration of DMSO) for 45 min at 37°C and then placed in tonometers (Eschweiler, Kiel, Germany) to equilibrate at the requisite O_2_ tension before flux measurement (still in N‐MBS). Tonometers were flushed with warm, humidified gas mixtures, supplied at the appropriate O_2_ tension using a Wösthoff gas mixing pump (Speake et al. 1997). For flux measurements, RBC suspensions were then diluted 10‐fold into flux tubes, still equilibrated at the required O_2_ tension. Where its effects were investigated, tonometers and flux tubes also contained 0.1–1 mmol·L^−1^ GBT1118. For experiments involving A23187, the ionophore (6 *μ*mol·L^−1^ final) was added to warm saline at 37°C in test tubes while vortexing. For experiments involving *N*‐ethylmaleimide (NEM), NEM (1 mmol·L^−1^) was present during preincubation with GBT1118 after which aliquots were again diluted 10‐fold into flux tubes. For CLT, dissolved in DMSO, appropriate controls were all treated with the same concentration of solvent (0.1% final).

### Measurements of O_2_ affinity and RBC sickling

Hemoglobin O_2_ dissociation curves and RBC sickling were simultaneously evaluated using a Hemox analyzer (TCS Scientific). Whole blood was incubated for 60 min at 37°C with various concentrations of GBT1118 (in 2% DMSO) followed by transfer to the sample chamber of the Hemox analyzer. For RBCs untreated with GBT1118, blood was incubated with 2% DMSO. The blood samples were then saturated with compressed air and flushed with pure N_2_ to deoxygenate in the Hemox analyzer. The absorbance at wavelengths that correspond to the isosbestic point (570 nm) and deoxy‐Hb (560 nm) were recorded as a function of partial pressure of O_2_. During deoxygenation, RBCs were harvested from the sample chamber at 150, 40, 30, 20, 10, and 1.6 mmHg O_2_ and immediately fixed in deoxygenated phosphate‐buffered saline (PBS) containing 2% glutaraldehyde. The fix solution (PBS/2% glutaraldehyde) was deoxygenated by bubbling 100% N_2_ through the solution for 20 seconds. Images of fixed RBCs were captured using a light microscope at 40x magnification and sickled cells were quantitated by manual counting. Elongated RBCs with tapering of opposite ends that culminated in a point as well as nondiscoid RBCs with spiky turns were counted as sickled. Results were reported as % sickled cells, calculated as the number of sickled RBCs divided by the total number of RBCs multiplied by 100.

### Measurement of RBC water content

The effect of GBT1118 (45 min preincubation) was investigated on RBC volume, in cells incubated in Cl‐MBS under full oxygenation or deoxygenation for 60 min at 37°C. RBC water content was measured by the wet weight – dry weight method (Borgese et al. 1991). In brief, after incubation for 60 min at 5% Hct, RBCs were pelleted by centrifugation at 12,000 g for 15 min at 4°C. The extruded pellet was weighed immediately (to an accuracy of 0.01 mg) and again after drying for 18 h at 95°C. Water content was expressed as ml water per g dry cell solids (ml/g d.c.s.).

### K^+^ flux measurements

To determine the activity of the K^+^ transport pathways, K^+^ influx was measured at 37°C using ^86^Rb^+^ as a congener for K^+^ (Dunham and Ellory 1981; Hannemann et al. 2011). RBCs washed in N‐MBS, preincubated without or with GBT1118 for 45 min, equilibrated in tonometers for 20 min, and then diluted 10‐fold into saline, also preequilibrated at the appropriate O_2_ tension, at 260 mOsm·kg^−1^ and pH 7. Hypotonic swelling and low pH were chosen in order to stimulate the K^+^‐Cl^−^ cotransporter (KCC) (Gibson and Ellory 2003). ^86^Rb^+^ was added in 150 mmol·L^−1^ KNO_3_ to give a final [K^+^] of 7.5 mmol·L^−1^ in all experiments except those with HK saline and A23187‐treated RBCs. Typically, three flux conditions were used: (1) Cl‐MBS, (2) Cl‐MBS with clotrimazole (CLT), and (3) N‐MBS with CLT. After incubation with radioisotope for 10 min, RBCs were washed five times in ice‐cold Mg‐MBS to remove extracellular ^86^Rb^+^. Except for measurement of the Na^+^/K^+^ pump activity, ouabain (100 *μ*mol·L^−1^) and bumetanide (10 *μ*mol·L^−1^) were present in all experiments to obviate any K^+^ transport through the Na^+^/K^+^ pump and the Na^+^‐K^+^‐2Cl^−^ cotransporter, respectively. Gardos channel activity was determined as the CLT‐sensitive (5 *μ*mol·L^−1^) K^+^ influx (using conditions 1 & 2); KCC activity as the Cl^−^‐dependent K^+^ influx (using flux conditions 2 & 3); and P_sickle_ as the deoxygenation‐induced, CLT‐insensitive K^+^ influx measured in the absence of Cl^−^ (condition 3). Na^+^/K^+^ pump activity was determined as the ouabain‐sensitive K^+^ influx. When measuring Gardos channel activity in the presence of A23187, a K^+^ uptake measurement was carried out with serial samples of RBCs taken at 1, 3, 5, 7, 10, and 15 min after addition of ^86^Rb^+^. For these experiments, extracellular [Ca^2+^] was 10 *μ*mol·L^−1^ which given a Donnan ratio of 1.43 would give an intracellular [Ca^2+^] of about 20 *μ*mol·L^−1^. Aliquots were added directly into ice‐cold Mg‐MBS that was layered over dibutyl phthalate oil (0.4 mL) whose density is such that on centrifugation (16,000 g, 15 sec) the oil allows the passage of RBCs only. The tube interior surface above the oil was carefully washed twice with ice‐cold Mg‐MBS, before the remaining oil was removed. For all flux measurements, the RBC pellet was lysed with Triton X‐100 (0.1%) and protein precipitated with trichloroacetic acid (5%). Activity was then measured as Čerenkov radiation by liquid scintillation (Packard Tri‐Carb 2800TR, Perkin Elmer). Either microhematocrit determination or the cyanohemoglobin method was used to measure the Hct with appropriate samples for this taken before the start of each experiment. There was minimal (<1%) hemolysis in all experiments.

### Statistics

**Figure 2:**
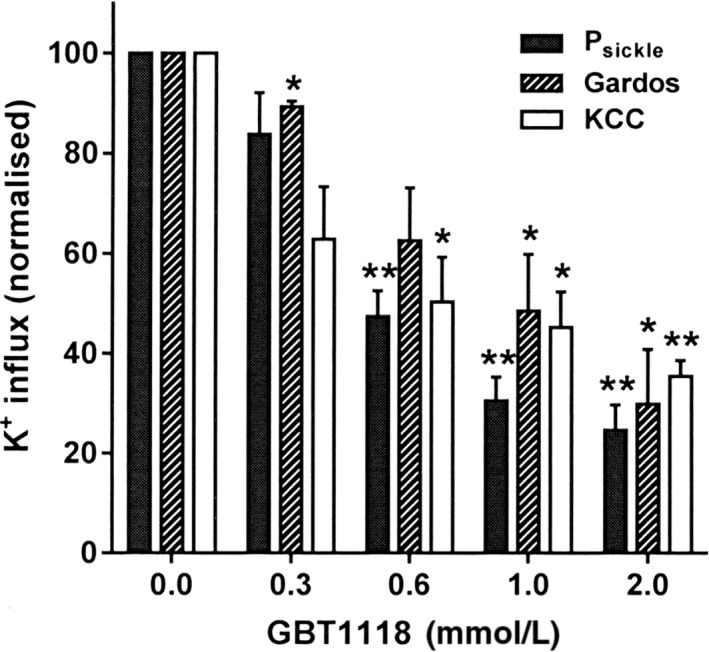
Effect of GBT1118 on the cation permeability of RBCs from patients with SCA. RBCs were preincubated for 45 min without or with different concentrations of GBT1118 (0, 0.3, 0.6, and 1 mmol·L^−1^) at 20% Hct at 37°C after which aliquots were equilibrated in Eschweiler tonometers for 20 min at 0 mmHg O_2_. Cell suspensions were then diluted 10‐fold into test tubes for measurement of K^+^ influxes: P_sickle_ activity was defined as the K^+^ influx in N‐MBS in the presence of clotrimazole (5 *μ*mol·L^−1^), given in mmol K^+^·(L cells.h)^−1^; Gardos channel activity as the clotrimazole (5 *μ*mol·L^−1^)‐sensitive K^+^ influx in Cl‐MBS; and KCC activity as the K^+^ influx in the presence and absence of Cl^−^, with 5 *μ*mol·L^−1^ clotrimazole. All fluxes were measured over 10 min at an extracellular K^+^ of 7.5 mmol·L^−1^ and are given normalized to their value in control RBCs untreated with GBT1118. For treated red cells, GBT1118 was present throughout flux measurement. Ouabain (100 *μ*mol·L^−1^) and bumetanide (10 *μ*mol·L^−1^) were also present for all influx measurements. Histograms represent means ± S.E.M., *n* = 3. *P <* *0.05; ** *P *<* *0.01.

Results are presented as means ± S.E.M. of n observations in RBC samples taken from different individuals. Where appropriate, comparisons were made using paired student t tests. When nonelectrolyte hemolysis was measured over time, multiple t tests followed by Holm*‐*Šídák corrections for multiple comparisons were used (Graphpad Prism 6; CA, USA). Correlations in Figure 2C were made using the Pearson correlation test. The level of significance used was *P *<* *0.05.

## Results

### The effect of GBT1118 on oxygen affinity, RBC sickling, and cation permeability

In the preliminary set of experiments, the effect of GBT1118 on O_2_ saturation and sickling was confirmed in whole blood from SCA patients (Fig. 1A and B). The P_50_ of untreated blood was about 31 mmHg, in agreement with previously published values (Milner 1974). When whole blood (20% hematocrit) was incubated with GBT1118 at increasing concentrations (0.3, 0.6, and 1.0 mmol·L^−1^), the P_50_ reduced progressively to 24, 9, and 4 mmHg, respectively (Fig. 1A). As expected, GBT1118 also reduced hypoxia‐induced sickling (Fig. 1B). In the absence of GBT1118, 50% of the total RBC population were sickled at an O_2_ tension of 30 mmHg. In contrast, relatively lower O_2_ tensions of 20, 8, and 4 mmHg were required to achieve 50% sickling in the presence of GBT1118 (0.3, 0.6, and 1.0 mmol·L^−1^, respectively), (Fig. 1B). The effect of different concentrations of GBT1118 was also measured on the main cation transporters of sickle cells. As the concentration of GBT118 was increased, P_sickle_, Gardos channel, and KCC were all progressively inhibited (shown in Fig. 2 for 0 mmHg O_2_). From these experiments, a concentration of 1 mmol·L^−1^ was selected to investigate further the effects of GBT1118 on RBC phenotype. Limited experiments were also carried out on normal RBCs from HbAA individuals, preincubated without or with GBT1118 (1 mmol·L^−1^) for 45 min. In RBCs pharmacologically loaded with Ca^2+^ (10 *μ*mol·L^−1^ extracellular) Gardos channel, after 15 min, activity declined from 58.7 ± 1.5 mmol K^+^·(L cells)^−1^ in the absence of GBT1118 to 40.8 ± 0.02 in its presence. KCC activity was 0.60 ± 0.01 in the absence of GBT1118 and 0.20 ± 0.02 in its presence (10% swollen, pH 7.0). GBT1118 also inhibited the Na^+^/K^+^ pump with ouabain‐sensitive K^+^ influx declining from 2.11 mmol K^+^·(L cells)^−1^ in cells untreated with GBT1118 to 0.73 in its presence.

### The effect of GBT1118 on the activities of P_sickle_ and the Gardos channel in RBCs from SCA patients

The effects of GBT1118 (1 mmol·L^−1^) were investigated on the main cation pathways mediating RBC dehydration at three O_2_ tensions: fully oxygenated RBCs at 100 mmHg, fully deoxygenated RBCs at 0 mmHg, and RBCs at an intermediate O_2_ tension of 20 mmHg.

**Figure 3:**
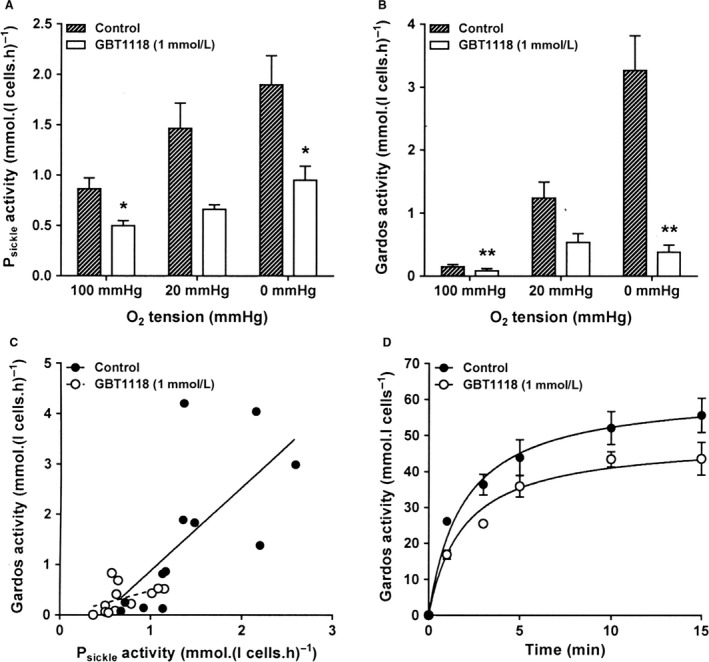
Effect of GBT1118 on the activities of cation channels in RBCs from patients with SCA. RBCs were preincubated without or with GBT1118 (1 mmol·L^−1^) in N‐MBS at 37°C for 45 min at 20% haematocrit. For treated RBCs, GBT1118 was present throughout all subsequent steps. RBCs were then equilibrated in Eschweiler tonometers for 20 min at the required oxygen tension, after which aliquots were diluted 10‐fold into flux tubes. For treated red cells, GBT1118 was present throughout flux measurement. Ouabain (100 *μ*mol·L^−1^) and bumetanide (10 *μ*mol·L^−1^) were also present for all influx measurements.(A) P_sickle_ activity, at an extracellular [K^+^] of 7.5 mM and defined as the K^+^ influx in N‐MBS in the presence of clotrimazole (5 *μ*mol·L^−1^), was measured over 10 min and given in mmol K^+^·(L cells.h)^−1^. Histograms represent means ± S.E.M., *n* = 4. * *P *<* *0.05. (B) Gardos channel activity, at an extracellular [K^+^] of 7.5 mmol·L^−1^ and defined as the clotrimazole (5 *μ*mol·L^−1^)‐sensitive K^+^ influx in Cl‐MBS, was measured over 10 min and given in mmol K^+^·(L cells.h)^−1^. Histograms represent means ± S.E.M. *n* = 4. **P <* *0.01. (C) Correlation of the activities of P_sickle_ and Gardos channel. Pearson correlation coefficients were *r* = 0.675 (*P *<* *0.02) and *r* = 0.46 (*P *=* *0.13) in the absence and presence of GBT1118, respectively. (D) Gardos channel activity in Ca^2+^ loaded RBCs. After preincubation without or with GBT1118, RBCs were suspended in air in HK‐MBS containing 10 *μ*mol·L^−1^ CaCl_2_, and equilibrated in test tubes in the presence of the Ca^2+^ ionophore A23187 (6 *μ*mol·L^−1^) for 15 min. Gardos channel activity was measured, as for Figure 3B but at an extracellular [K^+^] of 80 mmol·L^−1^, at the time points indicated over 15 min and given in mmol K^+^·(L cells)^−1^. Symbols represent means ± S.E.M., *n* = 5. The curves are fitted by nonlinear regression using Graphpad Prism software which gave an apparent V_max_ of 59.9 ± 0.4 mmol K^+^·(L cells)^−1^ in the absence of GBT1118 and 52.1 ± 3.4 mmol K^+^·(L cells)^−1^ in its presence, and a half time of 1.6 ± 0.4 min and 2.5 ± 0.6 min, respectively, with the curves being significantly different (*P *<* *0.0001).

The effect of GBT1118 on the activities of the two conductive channels, P_sickle_ and the Gardos channel, is shown in Figure 3A–D. At all three O_2_ tensions, there was significant inhibition of both channels, reaching inhibitions of 48 ± 9% and 89 ± 4% for P_sickle_ and Gardos channel activities under fully deoxygenated conditions, and 51 ± 12% and 50 ± 16% at 20 mmHg (Fig. 3A and B). When the two channels were compared, there was significant correlation of their activities in untreated RBCs (Fig. 3C, *P* < 0.02). This correlation agrees with the postulate that entry of Ca^2+^ via P_sickle_ results in subsequent Gardos channel activation. In the presence of GBT1118, correlation was lost (*P *=* *0.13), probably as channel inhibition reduced the signal‐to‐noise ratio.

We also tested whether GBT1118 might affect the Gardos channel directly using pharmacological elevation of RBC [Ca^2+^]_i_ by means of the ionophore A23187 (6 *μ*mol·L^−1^) (Fig. 3D). In the presence of GBT1118, Gardos channel activation was still reduced in Ca^2+^‐loaded RBCs. The V_max_ and t_1/2_ in the absence and presence of GBT1118 were 59.9 ± 0.4 and 52.1 ± 3.4 mmol K^+^(L cells.h)^−1^, and 96 ± 24 and 150 ± 36 sec, respectively, indicative of a direct effect of GBT1118 on the channel (*P *<* *0.001).

### The effect of GBT1118 on the activity of the KCl cotransporter in RBCs from SCA patients

**Figure 4:**
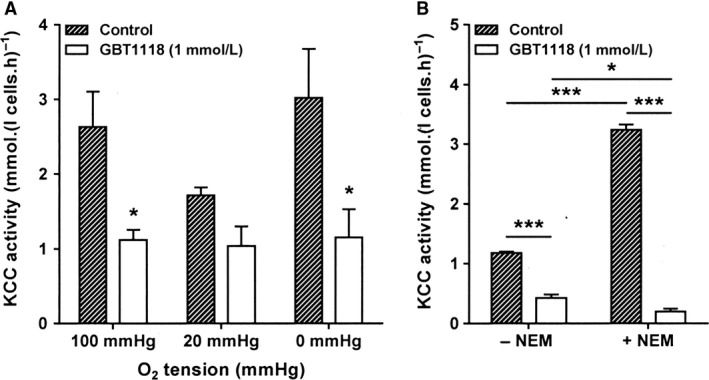
Effects of GBT1118 on the activity of the KCl cotransporter (KCC) in RBCs from patients with SCA. RBCs were preincubated without or with GBT1118 in N‐MBS, as described in the legend to Figure 3. They were then equilibrated in Eschweiler tonometers for 20 min at the required oxygen tension, after which aliquots were diluted 10‐fold into flux tubes containing either N‐MBS or Cl‐MBS. For treated red cells, GBT1118 was present throughout flux measurement. Ouabain (100 *μ*mol·L^−1^), bumetanide (10 *μ*mol·L^−1^), and clotrimazole (5 *μ*mol·L^−1^) were present for all influx measurements. (A) KCl cotransport (KCC) activity, at an extracellular [K^+^] of 7.5 mM and defined as the K^+^ influx in the presence and absence of Cl^−^, was measured over 10 min and given in mmol K^+^·(L cells.h)^−1^. Histograms represent means ± S.E.M. for RBCs from *n* = 4 patients. *P <* *0.05. (B) KCC activity in *N*‐ethylmaleimide (NEM)‐treated RBCs. RBCs were first preincubated in N‐MBS without or with either or both GBT1118 (1 mM) and *N*‐ethylmaleimide (NEM, 1 mmol L^−1^) for 45 min at 37°C, 20% Hct. RBCs were then diluted 10‐fold into flux tubes, with either or both GBT1118 and NEM present as required, and KCC activity measured at an extracellular [K^+^] of 7.5 mmol L^−1^, as in the legend to Figure 4A, over 10 min and given in mmol K^+^·(L cells.h)^−1^. Histograms are means ± S.E.M., *n *=* *5. *P < 0.05, ***P <* *0.001.

The third transporter involved in dehydration of sickle cells is the KCC. The effect of GBT1118 was again investigated on this system. At all three O_2_ tensions, KCC activity was reduced, significantly under fully oxygenated and fully deoxygenated conditions, with inhibitions of 55 ± 6% and 62 ± 13%, respectively (Fig. 4A). Inhibition was also observed at the intermediate O_2_ tension (40 ± 16%) (Fig. 4A, *P* < 0.05 in normalized fluxes).

To investigate whether GBT1118 had its effect on KCC activity via the regulatory protein kinase and phosphatase enzymes or directly on the transporter, its action was examined in RBCs pretreated with NEM. NEM is thought to act by preventing the activity of an inhibitory protein kinase upstream from the transporter, thereby maximally stimulating KCC activity. In both control cells untreated with NEM and those pretreated with NEM (1 mmol·L^−1^, for 45 min), GBT1118 produced significant inhibition (Fig. 4B). These findings are consistent with an action of GBT1118 at least in part directly on the KCC transport protein. Similar findings were observed in normal RBCs from HbAA individuals in which NEM‐stimulated KCC activity declined from 1.500 ± 0.007 in the absence of GBT1118 (1 mmol·L^−1^) to 0.200 ± 0.020 in its presence.

### The effect of GBT1118 on volume of RBCs from SCA patients

**Figure 5:**
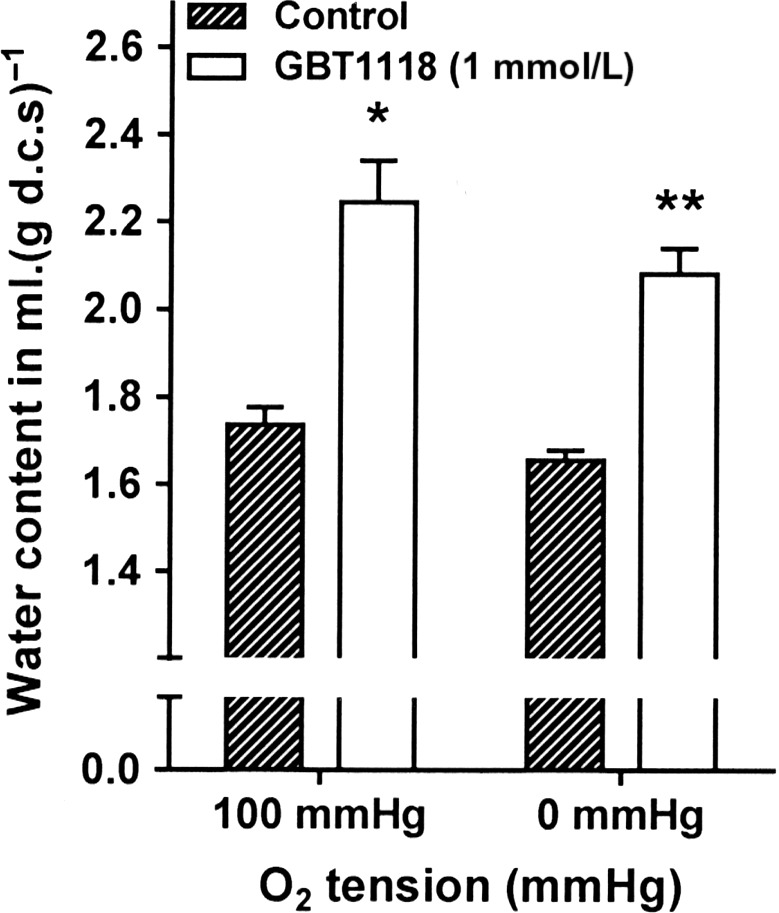
Effects of GBT1118 on cell volume of in RBCs from patients with SCA **.** RBCs were preincubated without or with GBT1118 (1 mmol·L^‐1^) in N‐MBS, as described in the legend to Figure 3. Aliquots were then incubated at pH 7 in Cl‐MBS for 60 min under fully oxygenated (100 mmHg O_2_) and fully deoxygenated conditions (0 mmHg O_2_). Cell volume was then measured by wet weight minus dry weight and expressed as ml water per g dry cell solids [mL (g d.c.s.)^−1^] (Borgese et al. 1991). Histograms are means ± S.E.M., *n *=* *5. *P < 0.05, **P < 0.01.

Through its inhibitory effects on all three cation pathways involved in dehydration of sickle cells, GBT1118 would be expected to maintain RBC hydration. This was investigated under fully oxygenated and fully deoxygenated conditions (Fig. 5) at pH 7 to be consistent with experiments on transport pathways. Results showed that GBT1118 significantly increased RBC hydration at 100 and 0 mmHg O_2_ (*P *<* *0.02 and *P *<* *0.002, respectively). Its inhibitory effects on the dehydrating ion pathways provide an explanation for this although an effect on water channels, for example, AQP1, cannot be excluded.

### The effect of GBT1118 on hemolysis of RBCs from SCA patients

**Figure 6:**
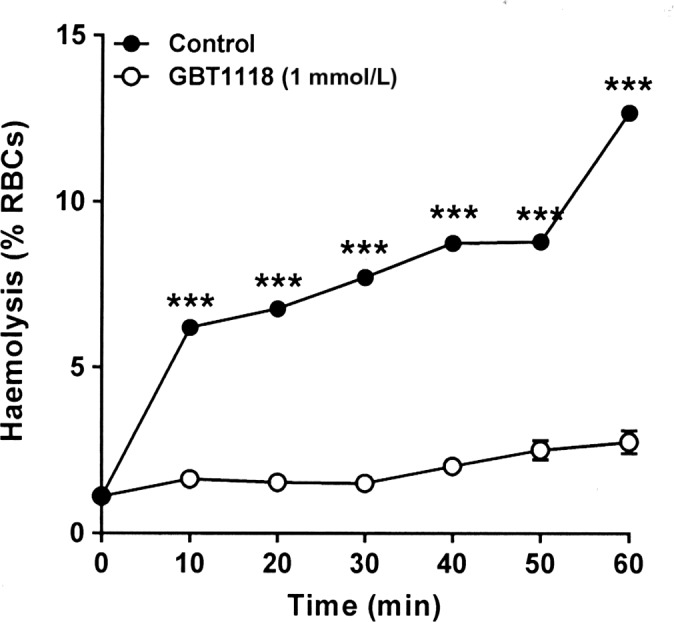
Effect of GBT1118 on hemolysis of RBCs from patients with SCA in deoxygenated isosmotic sucrose solutions. RBCs were preincubated without or with GBT1118 in Ca^2+^‐free N‐MBS at 37°C for 45 min. They were then incubated in isosmotic sucrose solution in Eschweiler tonometers and equilibrated with nitrogen for 60 min. For treated red cells, GBT1118 was present throughout flux measurement. Hemolysis was determined by removing serial aliquots of RBC suspension, pelleting intact RBCs, measuring optical density of the supernatant at 540 nm and was expressed as a percentage of total hemolysis, as determined by lysing a RBC aliquot using Triton X‐100 (0.1%). Data points represent means ± S.E.M., *n* = 5. ***P <* *0.001.

The final series of experiments examined the action of GBT1118 on an RBC hemolysis assay. In this assay, RBCs are deoxygenated in isosmotic sucrose solutions and incubated for up to 60 min. In RBCs pretreated with GBT1118, there was significant inhibition of RBC hemolysis from 10 min onwards, reaching a level of 78 ± 3% after 60 min (Fig. 6). Taken together, GBT1118 appears to stabilize the RBC membrane as well as increasing O_2_ affinity, reducing sickling and inhibiting the major cation transport pathways.

## Discussion

GBT1118 is an analogue of voxelotor (GBT440) with similar pharmacological properties. It was used here to investigate its effect on the RBCs transporter systems associated with cell dehydration. In whole blood, GBT1118 elicits a marked reduction in the P_50_ for O_2_ saturation being several fold more active than 5HMF. There is, concomitantly, a marked reduction in RBC sickling. A compound with such properties combining antisickling activities as well as beneficial effects on RBC hydration may well be effective in ameliorating complications in SCA patients.

The present results are significant because they show that GBT1118, in addition to its effect on O_2_ affinity and sickling, has a marked inhibitory effect on the three cation pathways most commonly associated with RBC dehydration (Lew and Bookchin 2005): P_sickle_, the Gardos channel, and the KCC. These effects were more pronounced at concentrations (1 mM) higher than that needed for antisickling activities (0.3 mmol·L^−1^). The correlation between activities of P_sickle_ and the Gardos channel, at least in RBCs untreated with GBT1118, indicate that part of its effect is mediated by decreased Ca^2+^ entry through the nonspecific cation conductance activated by HbS polymerization and the sickling shape change. In addition, however, GBT1118 was also an effective inhibitor of the Gardos channel in RBCs pharmacologically loaded with Ca^2+^, conditions which circumvent any requirement for Ca^2+^ entry via P_sickle_. In the presence of GBT1118, Gardos channel V_max_ was reduced and t_1/2_ increased.

The activity of KCC, the third cation transporter implicated in RBC dehydration, was also markedly inhibited by GBT1118. This transporter is around 50 times more active in sickle cells compared with RBCs from normal individuals (Brugnara et al. 1986). In RBCs from normal HbAA individuals, KCC activity is very low or absent by the time young RBCs have matured (Hall and Ellory 1986), and it becomes refractory to the usual physiological stimuli such as increased volume or low pH. As it remains amenable to pharmacological activation, this maturation is presumably through loss of its regulatory protein kinase/phosphatase cascade (Gibson and Ellory 2003), while the transporter remains present in the RBC membrane.

The activity of KCC in sickle cells also shows an abnormal O_2_ dependence (Gibson et al. 1998). In normal RBCs, the transporter is monotonically inhibited as O_2_ tension is reduced with peak activity observed in fully oxygenated RBCs, and a P_50_ for O_2_ close to that of O_2_ binding to Hb. The mechanism by which O_2_ has its effects has recently been elucidated by the work of Low and colleagues (Chu et al. 2016; Zheng et al. 2018), who propose that enhanced binding of deoxygenated Hb to band 3 displaces glycolytic and other enzymes, notably WNK1, leading to inhibition of the transporter. In sickle cells, as O_2_ levels are reduced, the activity of KCC goes through a nadir at about half saturation of Hb for O_2_ (Gibson et al. 1998). At lower O_2_ tensions, activity increases again such that in fully deoxygenated RBCs activity is similar if not slightly higher than that under fully oxygenated conditions. This O_2_‐dependent behavior was confirmed in the present work. Nevertheless, GBT1118 inhibited KCC activity at the three O_2_ tensions investigated. In addition, GBT1118 remained active in NEM‐treated RBCs, indicative of a direct inhibitory effect on the transporter, rather than through the regulatory phosphorylation.

Consistent with marked inhibitory action on the dehydrating ion pathways, GBT1118 was found to protect RBC hydration under fully oxygenated and fully deoxygenated conditions. Similar effects on cation transport and RBC hydration have been observed previously with the heterocyclic aldehyde 5HMF (Hannemann et al. 2014).

In the final series of experiments, GBT1118 was tested on an in vitro hemolysis assay (Browning et al. 2007; Milligan et al. 2013). RBCs were suspended in deoxygenated isosmotic sucrose solutions. Under these conditions, progressive hemolysis is observed in RBCs from SCA patients but not those from normal individuals (HbAA). GBT1118 showed marked protection against lysis, indicative of a reduction in RBC fragility.

The present work suggests that GBT1118 may have considerable protective effects on sickle RBCs. First, through increasing O_2_ affinity of HbS, reducing polymerization and sickling; second, through reduction in [HbS] which will also increase the lag time to HbS polymerization even if O_2_ levels fall sufficiently to precipitate eventual sickling; and, third, through stabilizing the RBC membrane and protecting against hemolysis.

## Conflict of Interest

DO and KD are employees of Global Blood Therapeutics. There are no other conflicts of interest.
